# Evaluation of Cholera Antigen-Specific Gut-Homing β7-Positive Antibody-Secreting Cells in the Systemic Circulation of Oral Cholera Vaccinees Receiving Doses at Different Intervals

**DOI:** 10.3390/vaccines13090919

**Published:** 2025-08-28

**Authors:** Polash Chandra Karmakar, Rasheduzzaman Rashu, Mohammad Rubel Hoq, Umme Salma, Kamrul Islam, Nusrat Jahan, Naoshin Sharmin Nishat, Aklima Akter, Sultana Rownok Jahan, Pinki Dash, Amit Saha, Edward T. Ryan, Firdausi Qadri, Taufiqur Rahman Bhuiyan

**Affiliations:** 1 icddr,b (International Centre for Diarrheal Disease Research, Bangladesh), Dhaka 1212, Bangladesh; polash.chandra@icddrb.org (P.C.K.); mrashu@uwo.ca (R.R.); mrh16f@fsu.edu (M.R.H.); umme.salma@abbott.com (U.S.); kamrulislam@utexas.edu (K.I.); jahann@macewan.ca (N.J.); naoshin.imm19@gmail.com (N.S.N.); aklima@ualberta.ca (A.A.); sultanarownok.jahan@lhsc.on.ca (S.R.J.); pinki.dash@icddrb.org (P.D.); asaha@kirby.unsw.edu.au (A.S.); fqadri@icddrb.org (F.Q.); 2 Division of Infectious Diseases, Massachusetts General Hospital, Boston, MA 02114, USA; etryan@mgh.harvard.edu; 3 Department of Medicine, Harvard Medical School, Boston, MA 02115, USA; 4 Department of Immunology and Infectious Diseases, Harvard School of Public Health, Boston, MA 02115, USA

**Keywords:** *Vibrio cholerae*, gut homing, antibody secreting cell, oral cholera vaccine, Shanchol

## Abstract

**Background/Objectives**: Cholera remains a significant global health challenge. Shanchol (ShantaBiotech, India), one of the WHO prequalified Oral Cholera Vaccines (OCVs) available until recently, has been used to immunize people as a two-dose regimen (14 days apart, on day 0 and 14). However, growing evidence suggests that a single-dose strategy may mediate short-term protection, especially in those over 5 years of age. Hence, it is crucial to design a suitable and effective administration scheme for Shanchol, particularly in cholera-endemic regions. **Methods**: In this study, adult volunteers were vaccinated with either a single dose, a two-dose regimen with a 14-day interval, or a two-dose regiment with a 30-day interval. We studied the antigen-specific helper memory (CD4+CD45RO+) and cytotoxic memory (CD8+CD45RO+) T cells responses of vaccinees along with the specific mucosal immune responses to gut-homing ß7 lipopolysaccharides (LPSs). **Results**: By day 7 post-vaccination, Shanchol induced robust helper and cytotoxic memory T cell responses to *V. cholerae* membrane protein (AKI-MP) following a single dose of vaccination. In the two-dose groups, we observed a significant elevation of AKI-MP-specific responses after the 2nd dose. We found that circulatory gut homing (β7+) LPS-specific IgA responses of antibody-secreting cells (ASCs) peaked at D7 among all vaccine groups. Moreover, we observed that β7+ LPS-specific ASCs at D7 significantly correlated with the LPS-specific antibody titer in plasma. **Conclusions**: These findings suggest that a single dose of OCV in adults induces immune responses comparable to a two-dose regimen, suggesting a single-dose vaccination may be adequate to mediate protection against cholera in cholera endemic zones—especially in reactive campaigns.

## 1. Introduction

Cholera, a debilitating diarrheal disease caused by *Vibrio cholerae*, remains a serious global challenge to public health. It causes an estimated 1.3 to 4.0 million cases per year and 21,000 to 143,000 deaths worldwide [1,2]. Among more than 200 serogroups, O1 and O139 are identified as the main causative agents of cholera [3], and O1 has been found responsible for most of the recent epidemic cases [4]. Serogroup O1 can be classified as classical and El Tor biotypes, and both biotypes are further classified into Ogawa and Inaba serotypes [5,6,7]. In addition to the immunogenic activities of *V. cholerae* exotoxin, studies suggest that OSPs (O-specific polysaccharides) and LPSs (lipopolysaccharides) can also induce mucosal immune responses, which make them the target components to provide treatment or protection against cholera [4]. This study focuses on Shanchol (Shanta Biotechnics, Hyderabad, India), one of the three WHO-prequalified Oral Cholera Vaccines (OCVs), to compare the dose-dependent immunity to LPSs.

Shanchol, an inactivated bivalent whole-cell vaccine, which contains inactivated *Vibrio cholerae* O1 and O139, was available in the OCV stockpile of the WHO until it was recently updated with Euvichol (EuBiologicals Co., Ltd., Seoul, Republic of Korea) [8]. Studies suggest that LPSs and OSPs are the protective antigens against cholera [4]. Earlier studies revealed dose-dependent T- and B-cell-mediated immunogenicity against cholera after administering another OCV, Dukoral (WC-rBS; Crucell, Stockhlom, Sweden), in both children and adults [9]. Using both single and two doses of Shanchol, studies have assessed the safety of the vaccine [10], the responses of mucosal and systemic antibodies along with memory B cell (MBC) responses [9]. However, vaccine-induced dose-dependent T cell memory response and subsequent antibody responses have not been reported yet.

Protection following natural infections or vaccination against non-invasive pathogens results primarily from antigen-specific antibody responses. Local responses at mucosal tissues often give protection to non-invasive mucosal infections, though this protection is not always reflected by the antibody secretion from distant tissues [4,11,12]. Therefore, the migratory and stimulated antibody-secretory cells (ASCs) from intestinal tissue [13], found in systemic circulation due to their migratory nature and retaining mucosal-homing markers like β7 [14], can be measured to understand the gut-homing efficacy after vaccination. Although the circulating ASC responses along with IgA and IgG secreting memory B cell (MBC) responses against OSPs have been studied on a two-dose vaccine regimen (Shanchol) [9], it is important to enumerate gut-homing β7 marker-specific ASCs to better understand the mucosal protection after vaccination using the oral cholera vaccine. Gut-homing ASCs are circulatory plasmablasts or plasma cells that can migrate to the gut to support gut immunity by producing antibodies [15]. Additionally, the term MBCs stands for the specialized B cells that can memorize the antigen of infectious agents and thus plays a crucial role in long-term immunity [16].

In the current study, we vaccinated sixty adult participants with Shanchol in Bangladesh in a single dose, two doses at a 14-day interval, and two doses at a 30-day interval to assess the efficacy level in terms of T cell activity and mucosal response. We measured the antigen-specific helper and cytotoxic memory T cell responses, *V. cholerae* LPS-specific gut-homing ASCs responses and LPS-specific systemic responses.

## 2. Materials and Methods

### 2.1. Study Groups and Recruitment of Participants

**Figure 1:**
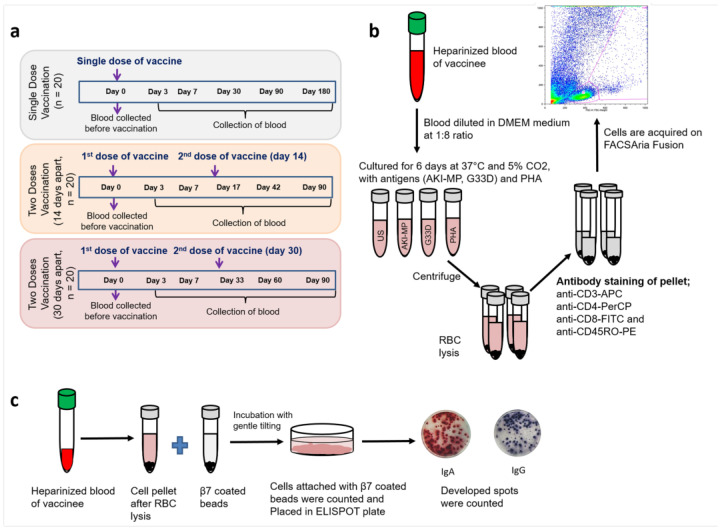
Blood collection schemes and flow cytometric assay of cell-mediated immune response in activated whole blood (FASCIA). (**a**) Oral cholera vaccine (OCV) Shanchol was given as a single dose (n = 20), two doses 14 days apart (n = 20), and two doses 30 days apart (n = 20). Blood was collected just before the vaccine administration on D0. (**b**) Illustration of FASCIA assay. In the figure, the words US, AKI-MP, G33D, and PHA represent “unstimulated”, “*V. cholerae* membrane protein”, “modified cholera toxin B subunit”, and “phytohemagglutinin”, respectively. (**c**) Illustration of gut-homing antibody secreting cells (ASCs) assay.

A total of 60 healthy adult participants (age: 18–45 years) were enrolled in the study. They were equally divided into 3 groups: (a) one dose of Shanchol, (b) two doses at a 14-day interval (vaccine administration at day 0 and day 14), and (c) two doses at a 30-day interval (at day 0 and day 30). Venous blood samples were collected in sodium-heparinized tubes on study days, depending on the vaccine administration (Figure 1a). The demographic characteristics of study participants including their age, sex, and ABO blood group types are mentioned in Table 1. We assessed the memory T cell response (both CD4 and CD8) of the vaccinees from each study day. Considering these T cell activities, a fraction of samples was also examined for gut-homing related lymphocytic responses and antibody responses against LPSs of *V. cholerae* O1.

### 2.2. T-Cell Proliferation Analysis

We performed a flow cytometric assay of cell-mediated immune response in the activated whole blood (FASCIA) technique described previously [17] to analyze T-cell responses to Shanchol. By incubating 50 µL of whole blood with the presence or absence of specific antigens, T cell responses and proliferation were measured. Briefly, blood (heparinized) was diluted with DMEM medium [DMEM F12 medium (Invitrogen, Carlsbad, CA, USA) with 10% fetal calf serum (HyClone, PerBio Science, Erembodegem, Belgium) and 0.5 mg/mL gentamicin (Gibco, New York, NY, USA)] at a 1:8 dilution and incubated with antigens at 37 °C and 5% CO_2_ in polystyrene tubes (BD, Franklin Lakes, NJ, USA). We used *V. cholerae* membrane protein (AKI-MP; 10 µg/mL) and modified cholera toxin B subunit (mCTB, named as G33D; 10 µg/mL) as stimulating antigens, and phytohemagglutinin (PHA; 10 µg/mL) was used as a positive control. The tube containing DMEM medium with no stimulating agents was considered as negative control. After 6 days, supernatants were removed, and red blood cells (RBCs) were lysed with ammonium chloride (NH_4_Cl) lysing solution. The remaining cells were stained with anti-CD3-APC, anti-CD4-PerCP, anti-CD8-FITC, and anti-CD45RO-PE antibodies (BD, USA) (Figure 1b). Stained cells were acquired on FACSAria Fusion (BD, San Jose, CA, USA) using the FACSDiva software (v8.0.2), and the gating strategies are shown in Figure S1. Frequencies of memory T cell types were analyzed with FlowJo software (version 10.6.1, TreeStar Inc., San Francisco, CA, USA).

AKI-MP was prepared by sonicating *V. cholerae* (O1, El Tor, Ogawa strain X25049) [18]. A mass spectrometry analysis of MP showed different bacterial antigens other than cholera toxin (CTB). Therefore, the assay focused on AKI-MP to relate the potentials of whole cell vaccines. Modified CTB antigen (mCTB), having one amino acid substitution (Gly-33→Asp; G33D), was also included to observe the ability of OCV to produce immunity against cholera toxin. mCTBs were found to have stronger FASCIA responses compared to unaltered CTBs and proved to have considerably diminished affinity for GM1 gangliosides [17]. So, mCTB was the antigen of choice for T-cell stimulation experiments.

### 2.3. Gut-Homing Antibody Secreting Cells (ASCs) Assay

A gut-homing ASC assay was carried out by following the protocol described earlier [14,19] with slight modifications. Briefly, in a 9 mL culture tube (Pyrex, Corning, PA, USA), anti-rat IgG beads (Dynabeads; Invitrogen, Oslo, Norway) were placed with 3 mL of cold fluorescence-activated cell sorter (FACS) buffer. For 1 mL of blood, 25 µL beads (~10^7^ beads) were used and placed in a magnet chamber (Dynal MPC-6; Oslo, Norway) for 2 min. The supernatant was removed without disturbing the tube from the magnet, and this washing process was repeated twice. After removing the tube from the magnet, beads were resuspended in 25 µL of FACS buffer. Then, 4 µL of rat anti-human integrin β7 (BD Pharmingen, Milpitas, CA, USA) was added to the beads, and the tubes were incubated for 30 min at 4 °C with gentle tilting. After that, the tubes were washed twice following the same washing procedure.

From heparinized 1 mL blood, after removing plasma (centrifugation at 600× *g* for 10 min), the remaining blood was transferred into a Falcon tube (BD, CA, USA) and mixed with 30 mL of 1 M NH_4_Cl lysing solution, and then it was incubated for 7 min to lyse red blood cells (RBCs) and centrifuged at 772× *g* for 5 min. Then, the supernatant was removed, the pellet was resuspended in 1 mL FACS buffer, and it was mixed with anti-human β7-coated beads as described above. This mixture was incubated at 4 °C for 45 min. Then, 3 mL of FACS buffer was added to the tubes, which were kept in the magnet chamber, and they were washed twice with FACS buffer. Finally, the cells were resuspended with RPMI (Roswell Park Memorial Institute) complete medium [RPMI 1640 medium (Gibco, Carlsbad, CA, USA); 10% FBS (HyClone, Logan, UT, USA); 1% L-glutamine (Life Technologies Corporation, Gibco, Grand Island, NY, USA); 1% penicillin–streptomycin (Life Technologies Corporation, Gibco); 1% Na-pyruvate (Life Technologies Corporation, Gibco) and 0.1% gentamycin]. Cells were then counted and placed onto the nitrocellulose-bottom plates, which were previously coated with *V. cholerae* LPS antigen and a mixture of affinity-purified goat antibodies to human Ig κ and λ light chains [4]. Overall, 2500 to 5000 cells were used in duplicate wells to determine the total immunoglobulin spots. For antigen-specific spots, 15,000 to 20,000 cells were used in a single well. A horseradish peroxidase (HRP)-dependent two-color procedure (3-amino-9-ethylcarbazole [AEC] and 5-bromo-4-chloro-3-indolylphosphate–nitroblue tetrazolium [BCIP-NBT] substrate) was applied to enumerate spots according to the protocol described previously [19] (Figure 1c).

### 2.4. LPS-Specific IgA and IgG Antibody Responses in Plasma

Enzyme-linked immunosorbent assay (ELISA), as described previously [4], was conducted using plasma samples of vaccinees to assess IgA and IgG antibody responses to LPSs. A total of 100 µL of plasma from each participant and LPSs (2.5 µg/mL) of *V. cholerae* O1 Ogawa or Inaba were used in ELISA to detect antigen-specific antibodies using secondary antibodies named horseradish peroxidase-conjugated rabbit anti-human IgA and IgG (dilution 1:1000; Jackson ImmunoResearch, West Grove, PA, USA) [9]. ELISA plates were read kinetically at 450 nm optical density (OD) where the maximal change rate in OD was calculated as milli-absorbance per minute (mabs/min) unit [4]. Data were normalized and obtained following the procedure described previously, where responder frequencies were defined as a ≥2-fold increase over the baseline [4].

### 2.5. Statistical Analysis

Graph preparation and data analysis were conducted using GraphPad Prism 5.0 (GraphPad Software, Inc., La Jolla, CA, USA). We used Wilcoxon signed-rank t tests to assess the statistical differences of responses within a group. LPS-specific circulatory IgA and IgG responses at two day points of each vaccine group were analyzed using a two-tailed unpaired *t*-test. The association of gut-homing ASC with plasma antibody responses was evaluated using the Spearman correlation coefficient. *p* values were two-tailed, and *p* ≤ 0.05 was considered statistically significant.

## 3. Results

### 3.1. Frequency of CD4+ and CD8+ Populations with Memory T Cells

To enumerate the levels of activated helper and cytotoxic T cells among the three groups of vaccinees, we stimulated lymphocytes with *V. cholerae* antigens (AKI-MP and G33D). Helper and cytotoxic T cells were gated as CD3+CD4+ cells and CD3+CD8+, respectively. CD4+ helper T cells and CD8+ cytotoxic T cells were further gated as CD4+CD45RO+ and CD8+CD45RO+, which are the fraction of the memory population of helper and cytotoxic T cells, respectively.

**Figure 2:**
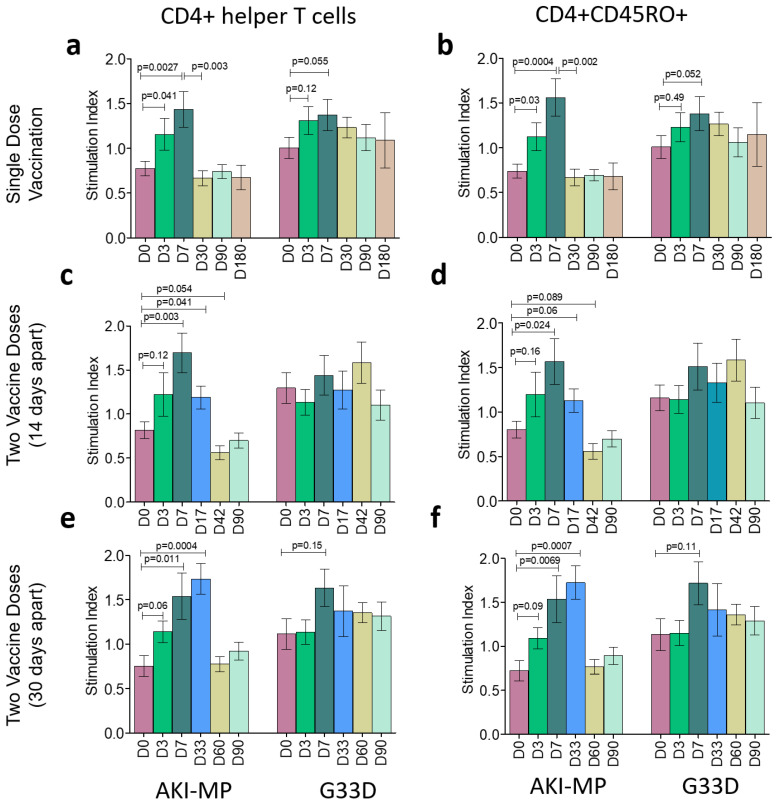
Helper T lymphocyte response in Shanchol vaccinees. Bar graphs represent the panel of helper T lymphocyte responses after stimulation with the *V. cholerae* membrane protein (AKI-MP) and modified cholera toxin B subunit (G33D) of three vaccine groups. CD4+ responses are shown among the vaccinees of a single dose (**a**), two doses with a 14-day interval (**c**), and two doses with a 30-day interval (**e**). Similarly, memory helper T cell (CD4+CD45RO+) responses are shown among the vaccinees of a single dose (**b**), two doses with a 14-day interval (**d**), and two doses with a 30-day interval (**f**). Stimulation indices are calculated as the ratio of the cell number responsive to antigen stimulation to its corresponding cell number in non-stimulation. Error bars represent standard errors of the means (mean ± SEM) with a 95% confidence interval. *p* ≤ 0.05 represents statistically significant differences from baseline levels (D0).

The significant increase or decrease in T cell populations at different study points was compared with D0 (before vaccination). In case of CD4+ response, participants having a single dose of vaccine (n = 20) showed a significant increase in cell population on D3 and D7 while stimulated with AKI-MP antigens (Figure 2). Responses decreased at subsequent time points (D30, D90, D180). CD4+ cells did not show any significant changes after G33D stimulation on different day points (Figure 2a). In this vaccine group, CD4 memory (CD4+CD45RO+) cells showed similar expression; they were elevated at D3 and D7 with AKI-MP stimulation, which decreased on subsequent days. No significant change was observed with G33D stimulation (Figure 2b). Participants (n = 20), who had two doses of vaccine with a 14-day interval, showed a significantly higher expression of CD4+ cells only at D7 and D17 (3 days after the 2nd dose), but the memory CD4+ expression rose only at D7 with AKI-MP stimulation. No remarkable change was observed in G33D stimulation (Figure 2c,d). Additionally, vaccinees (n = 20) of 30-day-interval doses showed a significantly higher expression of both CD4+ and CD4+CD45RO+ cells only at D7 and D33 (3 days after the 2nd dose) with AKI-MP stimulation. G33D stimulation showed no change similar to that observed in other groups of vaccinees (Figure 2e,f).

**Figure 3:**
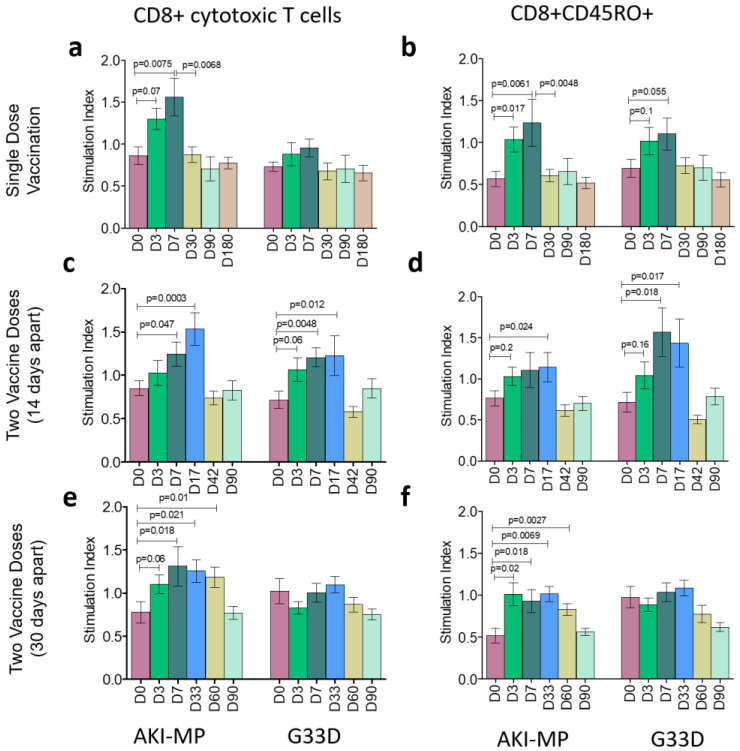
Cytotoxic T lymphocyte response in Shanchol vaccinees. Bar graphs represent the panel of cytotoxic T lymphocyte responses of the three vaccine groups after stimulation with *V. cholerae* membrane protein (AKI-MP) and modified cholera toxin B subunit (G33D). CD8+ responses are shown as (**a**) single dose, (**c**) two doses with a 14-day interval, and (**e**) two doses with a 30-day interval vaccine groups. Similarly, (**b**,**d**,**f**) represent memory cytotoxic T cell (CD8+CD45RO+) responses among the vaccinees of a single dose, two doses with a 14-day interval, and two doses with a 30-day interval, respectively. The responsive cell number after antigen stimulation, with its corresponding cell number to non-stimulation, indicates the stimulation index. Error bars show standard errors of the means (mean ± SEM). *p* ≤ 0.05 represents statistically significant differences from baseline levels (D0).

We also assessed cytotoxic T cell responses among all three vaccine groups, and we observed similar profiles to CD4+ T cell responses. With AKI-MP antigens, participants with a single dose showed a significant elevation of both CD8+ and CD8+CD45RO+ populations on D3 and D7, but no change was found in the case of G33D stimulation (Figure 3a,b). Interestingly, vaccinees of 14-day interval doses showed cytotoxic T cell stimulation against both AKI-MP and G33D antigens. CD8+ cells rose remarkably on D7 and D17 with AKI-MP and G33D, and they decreased on later days. Cytotoxic memory T cell populations also increased on D17 with AKI-MP and on D7 and D17 with G33D antigen (Figure 3c,d). Finally, among the group of 30-day-interval doses, we detected a significant increase in AKI-MP stimulated populations in the case of both CD8+ and CD8+CD45RO+ memory T cells on D7, D33, and D60, but no significant change was found for G33D (Figure 3e,f).

Moreover, we evaluated the stimulation of CD4+ and CD8+ T cell responses to *V. cholerae* membrane protein (AKI-MP) at D0 and D7 based on the ABO blood types of all of the vaccinees. We found significant rise in CD4+ T cell expression against AKI-MP in case of “O”, “A”, and “B” blood types (Figure S2a) and CD8+ T cell expression in all of the blood types (Figure S2b).

### 3.2. Total Gut-Homing LPS-Specific Immunoglobulin Responses Among the Vaccine Groups

The activities of both helper and cytotoxic T cells led us to examine the LPS-specific gut-homing response among vaccine groups (n = 10 for each group). So, we measured total gut-homing immunoglobulin-secreting cells (ISCs) through Enzyme Linked Immunospot (ELISPOT) assay from all groups at three time points: before the first vaccination, 7 days after the first dose, and 30 days after the last dose. Therefore, days selected for the single dose group were D0, D7, and D30, whereas D0, D7, and D42 were chosen for the 14-day-interval group. In case of a 30-day-interval group, we considered D0, D7, and D60 for this experiment.

**Figure 4:**
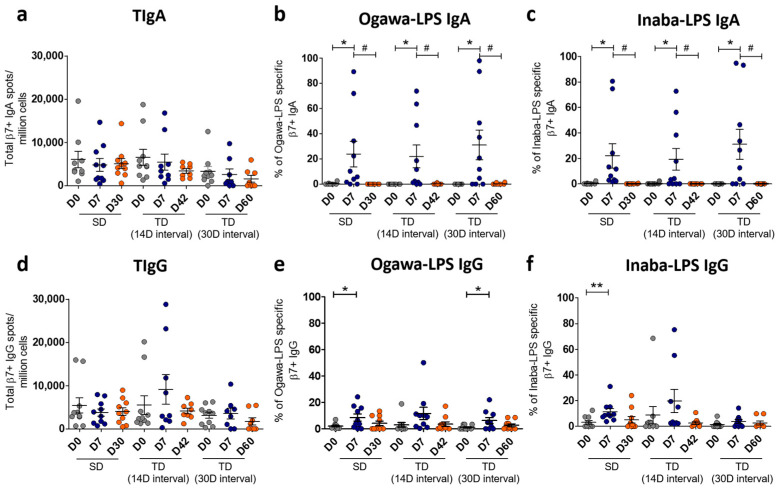
LPS-specific gut-homing antibody-secreting cell (ASC) responses in Shanchol vaccinees groups. Gut-homing (β7+) specific total immunoglobulin secreting cells are shown as (**a**) total IgA (TIgA) and (**d**) total IgG (TIgG). Percentages of LPS-specific β7+ ASC are represented as (**b**) Ogawa-specific IgA (Ogawa-LPS IgA) and (**c**) Inaba-specific IgA (Inaba-LPS IgA). Similarly, (**e**,**f**) stand for Ogawa-LPS IgG and Inaba-LPS IgG, respectively. The words SD means single dose and TD means two-dose vaccinees. Error bars show standard errors of the means (mean ± SEM). Asterisks (*) represent significant differences from baseline levels (D0), and (#) stands for significant differences of D7 responses to the later days. *, # = *p* < 0.05, ** = *p* < 0.01.

The number of total gut-homing immunoglobulin spots was calculated per million gut-homing (β7) cells at different day points of these three vaccine groups. In this study, spots of total immunoglobulin A (TIgA) did not show any difference among the day points of each vaccine group (Figure 4a). We found, in each vaccine group, a significant elevation of the percentages of Ogawa-LPS specific β7+ IgA secreting cells on D7, which decreased on later days (Figure 4b). Inaba-LPS-specific β7+ IgA secreting cells also showed similar expression patterns among all vaccine groups (Figure 4c). We also measured the expression of total immunoglobulin G (TIgG) among the groups, and no difference was observed (Figure 4d). The percentage of Ogawa-LPS-specific β7+ IgG secreting cells showed a significant rise on D7 of the single-dose and 30-day-interval groups. Unlike β7+ IgA secreting cells, no significant drop in the IgG population was found in later days (Figure 4e). Inaba LPS-specific β7+ IgG was found raised on D7 in the case of the single-dose group (Figure 4f). The percentages of either Ogawa or Inaba-specific IgG on later days were unchanged in all three vaccine groups.

Gut-homing Ogawa and Inaba LPS-specific β7+ IgA were also assessed at D0 and D7 based on the ABO blood types of all of the vaccinees. We observed a significant elevation of both Ogawa (Figure S3a) and Inaba (Figure S3b) LPS-specific β7+ IgA expression at D7 among the “O”, “A”, and “B” blood types.

### 3.3. Correlation of Gut-Homing ASCs and Circulatory Antibody Responses

**Figure 5:**
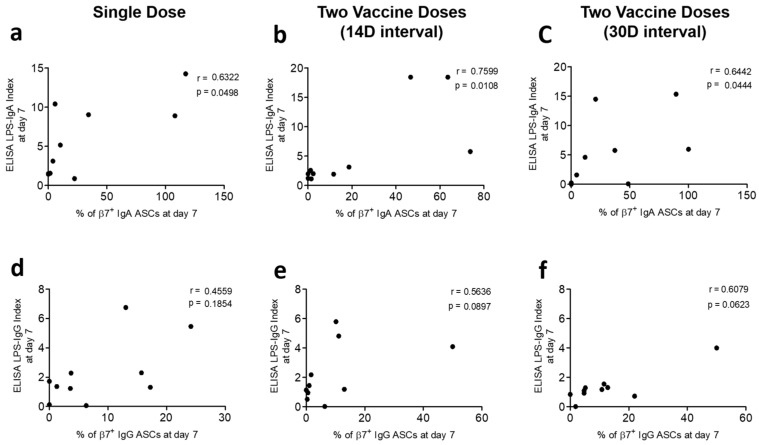
Association of gut-homing ASCs with circulatory antibody of D7. Spearman correlation of gut-homing (β7+) IgA secreting cells at D7 and LPSs (Ogawa)-specific circulatory IgA at D7 are represented in three vaccine groups: (**a**) single dose, (**b**) two dose (14-day interval), and (**c**) two dose (30-day interval). Similarly, β7+ IgG secreting cells (D7) and LPS (Ogawa)-specific circulatory IgG (D7) are shown among the (**d**) single-dose, (**e**) two-dose (14-day interval), and (**f**) two-dose (30-day interval) vaccine groups. The ELISA index is calculated as the ratio of the antibody level at D7 to its corresponding level at baseline (D0).

**Figure 6:**
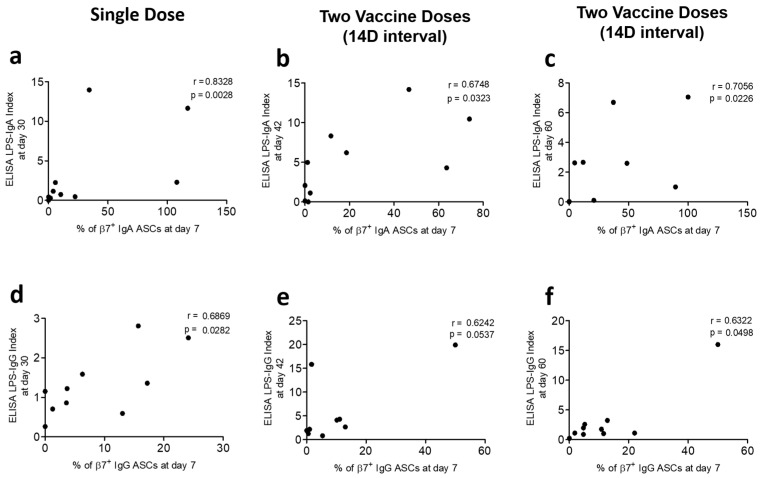
Association of gut-homing ASCs with circulatory antibody on day 30. Spearman correlations are shown of gut-homing (β7+) IgA secreting cells at D7 and LPS (Ogawa)-specific circulatory IgA at D30 of the (**a**) single-dose, (**b**) two-dose (14-day interval), and (**c**) two-dose (30-day interval) vaccine groups. Similarly, β7+ IgG secreting cells (D7) and LPS (Ogawa)-specific circulatory IgG at D30 are shown among three vaccine groups; (**d**) single dose, (**e**) two dose (14-day interval), and (**f**) two dose (30-day interval). The ELISA index is calculated as the ratio of the antibody level at D30 to its corresponding level at baseline (D0).

We determined the potential associations of gut-homing ASCs with antibody responses in plasma. We compared Ogawa LPS-specific IgA and IgG responses among β7+ ASCs on D7 with the corresponding circulatory antibody responses on D7 and subsequent days in each of the vaccine groups. We observed positive correlations of LPS-specific gut-homing β7+ IgA ASC responses with plasma LPS-specific IgA levels (ELISA indexes) at D7 and later days in all vaccine groups (Spearman r = 0.63 to 0.83; *p* = 0.0028 to 0.049) (Figure 5a–c and Figure 6a–c). In the case of β7+ IgG ASC responses at D7 in all vaccine groups, we did not find any correlation of LPS-specific IgG levels at D7 but observed positive correlations during subsequent days (Spearman r = 0.62 to 0.69; *p* = 0.028 to 0.05) (Figure 5d–f and Figure 6d–f).

As we observed increased Ogawa and Inaba LPS-specific β7+ IgA responses at D7 that declined at later day points for all vaccine groups, we further investigated the status of Ogawa LPS-specific immunoglobulins in plasma. We observed, among all the vaccine groups, Ogawa LPS-specific IgA responses in plasma being maximal at D7, and this response did not wane significantly during the later days (Figure S4a). Responses of Ogawa LPS-specific IgG in plasma were similar at D7 and later days (Figure S4b). A positive response was defined as a ≥2-fold increase over the baseline ratio and was defined as a responder.

## 4. Discussion

Since cholera continues as a significant health problem, the WHO recommends cholera vaccination for people living in active cholera transmission areas globally. Therefore, vaccination, two full doses or more recently a single dose, has been practiced since the last decade. After observing the devastating situation in Haiti in the 2010 epidemic along with in sub-Saharan Africa [20,21], the available OCVs, either Dukoral or Shanchol, and other OCVs that have become available, have been administered. Although Dukoral demonstrated 85% protective efficacy over six months after vaccination, a previous three-year field trial study in Bangladesh showed that this efficacy was over 50% among the Bangladeshi population [22]. Therefore, more affordable and easily administrable vaccines became part of several pilot studies among low-income countries.

Consequently, a killed whole-cell OCV named Shanchol was used to control cholera outbreak in Guinea, Haiti and South Sudan [23,24,25], and this oral vaccine is now considered as part of cholera prevention and control programs as several studies showed its 43% protective efficacy in children and 66% among Kolkata people in India [26]. There were Shanchol-related immunogenicity studies conducted in different countries [27,28] which conferred that the second dose of Shanchol gave a long immune protection compared to the first dose. Additionally, a Bangladeshi study on OSP-specific systemic, memory cell, and mucosal responses revealed that a single dose of Shanchol generated maximum responses without boosting with a second dose [9]. However, no evidence on LPS-related immunogenicity has been shown in the case of the Shanchol regimen with a single dose or two doses with different time intervals.

As an inactivated whole-cell vaccine, Shanchol is proposed to induce both OSP and LPS-specific responses. In a previous study conducted on Bangladeshi adults, we have already observed that a single dose of Shanchol is sufficient to produce maximal OSP-specific IgA, memory B cells, and mucosal responses [9]. In the current study, we included two separate dose schedules with 14-day and 30-day intervals, along with a single dose to set a better dose regimen of this vaccine, which might aid practical vaccination strategies in endemic regions. We have found LPS-specific responses of helper and cytotoxic memory T cells, and gut-homing signal, which correlated with the LPS-generated systemic responses, and this correlation is crucial for understanding long-term protection.

Bacterial LPSs and more specifically the OSPs, which are the immunogenic component of the LPSs, are the major components of the outer surface membrane of *V. cholerae*, which act as the prototypical endotoxin and induce strong stimulators of innate immunity. Therefore, studies on T cell-dependent antigens such as toxin-coregulated pilus (TcpA), LPSs, MP, and cytolysin/hemolysin (VCC) of *V. cholerae* had been observed among the Bangladeshi population [29]. That study showed a significant elevation of CD4+CD45RO+ at D7 after infection. In this study, we also have found a similar increase in CD4+ helper T cells and CD4+CD45RO+ memory T cells to *V. cholerae* membrane protein (AKI-MP) at D7 following vaccination. While using a single dose of Shanchol, maximal response was observed at D7, whereas two doses with 14-day and 30-day intervals showed significant retention of these elevated responses just after three days from the second vaccination (i.e., D17 and D33, respectively). We also checked the CD4+ and CD4+CD45RO+ responses of these three vaccine groups to modified cholera toxin B subunit (G33D), but no significant vaccine-induced change was observed on the following days. This vaccine can generate the T-cell immune responses among vaccinees to the MP of *V. cholerae*, which may play roles in protective immunity through B-cell responses and memory B-cell responses.

Cytotoxic T cells (CD8+) and cytotoxic memory T cells (CD8+CD45RO+) were also examined alongside the helper T cell responses, and we observed similar scenarios among these CD8+ populations. Remarkably higher CD8+ and CD8+CD45RO+ responses to AKI-MP were observed at day 7 post-vaccination among all of the vaccine groups. A similar rise in MP-related responses was found in the 14-day and 30-day interval groups following the second dose. If we summarize the data achieved from Shanchol-induced total T cell responses, we see high baseline responses to both AKI-MP and G33D (D0), which was similar to all vaccine groups in this study. Moreover, OCV Shanchol does not contain cholera toxin (CTB); hence, we were not supposed to obtain any T cell response against G33D. In this study, we did not observe any G33D-specific responses except some CD8+ T cell responses among the vaccinees of the 14-day interval dose group. This could possibly be due to the recurrent symptomatic or asymptomatic gut microbial infections in cholera-endemic areas, such as Bangladesh.

The *V. cholerae* O1-induced responses of gut-homing T-cell populations (CD4+ β7+ and CD8+ β7+) in circulation have already been studied in infected patients [30]. This study showed the elevation of gut-homing T and B cells with the involvement of CD8 cells along with the CD4-mediated cytokine responses (IFN-γ and IL-13). Additionally, experiments on the Shanchol vaccinees with 14-day interval doses have shown long-term activities of vibriocidal antibody and OSP-specific memory B cell responses. However, the level of OSP-specific IgA dropped after day 7 following the first dose of this vaccine [9]. Counting all of these, in the current study, we decided to scrutinize gut-homing marker (β7) expressing cells from circulation and measured LPS-specific antibody responses (for both Ogawa and Inaba) from these β7+ populations. We found significant raises of Ogawa-LPS and Inaba-LPS specific IgA antibodies at day 7 post-vaccination, which dropped remarkably in the later days. These were found in all of the vaccine groups, which means the second dose did not boost β7+ lymphocyte populations. However, IgG-secreting β7+ cells did not show a difference, because this cell type possibly stays for a longer period in the population of cholera-endemic areas.

We intended to observe the possibility of ABO blood type effects on the cellular responses. Our cellular data on T lymphocytes and gut-homing ASCs showed a significantly high elevation of responses at D7 compared to the baseline levels (D0). These effects were observed due to the 1st dose of the Shanchol vaccination (apart from the 2nd dose of the 14-day interval and 30-day interval). Therefore, while segregating the vaccinees based on their ABO blood types, we considered all sixty vaccinees and compared the data of D0 and D7. At D7, the significant rise of CD4+ and CD8+ T cells expression against AKI-MP in “O”, “A”, and “B” blood types indicated that the vaccinees of each blood group might have a similar T cell effect to specific antigens (see Figure S2). No significant change in responses was observed in the “AB” blood type, which was probably due to the low number of participants in that group. Moreover, different blood type specific gut-homing Ogawa and Inaba LPS-specific β7+ IgA responses showed a similar effect likewise with the T cell responses (See Figure S3). Therefore, we can conclude that ABO blood groups do not have any specific blood type effect on Shanchol-mediated cellular responses.

Accumulating the data generated from T lymphocytes and gut-homing ASC assays, we studied the response of the circulatory antibodies of all vaccine groups at D7 and later days. As the gut-homing LPS-specific IgA ASC responses of Ogawa and Inaba were similar, we scrutinized the potential associations of Ogawa β7+ ASC with its antibody responses in plasma. At D7, positive correlations were seen between LPS-specific IgA levels in plasma and β7+ IgA responses, which were practically reasonable. In addition, unlike β7+ IgA ASC responses, the plasma IgA persisted for subsequent days after D7. Therefore, we also saw positive correlations at later days with β7+ IgA ASC responses at D7 for all vaccine groups. In all vaccine groups, no potential correlation was found among β7+ IgG responses at D7 with the D7 responses of LPS-specific circulatory IgG. Like the IgG-secreting β7+ lymphocyte, this scenario can explain the sustainability of LPS-specific IgG in plasma among high-risk people. However, positive correlations of β7+ IgG responses at D7 develop with circulatory LPS-specific IgG at later days, which is evident of giving protection through IgG for a comparatively longer time period. Moreover, we plotted the responses of Ogawa LPS-specific circulatory IgA and IgG at D7 with later days and found no significant change in any vaccine group. Vaccine-generated protective immune response can persist with Shanchol whether it is administered as a single dose or a two-dose regimen.

This current study has some limitations. First, we conducted this study in Bangladesh, a country that is a cholera-endemic area, suggesting the possibility of previous *V. cholerae* exposure as well as immunological memory to the study participants. In this regard, it is not known whether OCV Shanchol-related responses in individuals in non-endemic areas would be similar to our findings. Second, since this study included adult individuals, OCV-generated LPS-induced immune protection has not been studied among children, although they are highly susceptible to cholera. Third, we enumerated the blood-derived LPS-specific gut-homing ASCs of the vaccinees, which may not directly correlate with the mucosal immune protection. Including immunohistochemical data would provide a more comprehensive understanding.

## 5. Conclusions

To the best of our knowledge and despite all these limitations, our study is the first attempt to examine Shanchol-induced immune protection regarding LPS-generated memory T lymphocyte response along with its gut-specific immunity among vaccinees of the single-dose and the full two-dose regimen. We observed that a single dose of this OCV can generate a continued LPS-induced immune response in circulation for up to 30 days, which may indicate a considerable correlate of protection against cholera infection. In the case of two-dose regimens, similar periods of protection were seen from the second dose, evidencing no boosting-related response. Therefore, a single dose of OCV Shanchol, applied to adults in a cholera-endemic area, may be sufficient to facilitate short-term protection. Single-dose OCV administration once or twice a year could maintain long-term protection. However, further studies with larger sample sizes are needed to assess the association between immune responses and period of protection, especially in children and populations from non-endemic zones for longer follow-up periods. Future studies assessing direct mucosal immunity will be crucial for developing widespread vaccination strategies.

## Figures and Tables

**Table 1:** Demographic characteristics of study participants.

Characteristics of Participants: (N = 60)				
		Single Dose (N = 20)	Two Doses with 14-Day Interval (N = 20)	Two Doses with 30-Day Interval (N = 20)
Age, [median (range)]		28.21 [22–45] years	27.33 [18–44] years	28.67 [18–42] years
Sex	No. (%) female	9 (45)	10 (50)	11 (55)
	No. (%) male	11 (55)	10 (50)	9 (45)
Blood type: No. (%)	O	4 (20)	8 (40)	9 (45)
	A	5 (25)	4 (20)	5 (25)
	B	8 (40)	7 (35)	5 (25)
	AB	3 (15)	1 (5)	1 (5)

## Data Availability

The raw data supporting the findings of this article will be made fully available by the authors without undue reservation.

## Supplementary Materials

The following supporting information can be downloaded at https://www.mdpi.com/article/10.3390/vaccines13090919/s1. Figure S1: Representative plot of fluorescence-activated cell sorting and gating strategy for memory CD4 and CD8 T lymphocytes. Figure S2: Blood group-specific Helper (CD4+) and Cytotoxic (CD8+) T lymphocyte response in Shanchol vaccinees. Figure S3: LPS-specific gut-homing antibody-secreting cell (ASC) responses in Shanchol vaccinees among different blood groups. Figure S4: LPS-specific Immunoglobulin A (IgA) and Immunoglobulin G (IgG) response to Ogawa in plasma.

## Abbreviations

The following abbreviations are used in this manuscript:

OCV	Oral Cholera Vaccine
LPS	Lipopolysaccharide
OSP	O-specific polysaccharide
MBC	Memory B cell
ASC	Antibody-secretory cell
FASCIA	Flow cytometric assay of cell-mediated immune response in activated whole blood
DMEM	Dulbecco’s Modified Eagle Medium
FACS	Fluorescence-activated cell sorter
PHA	Phytohemagglutinin
RPMI	Roswell Park Memorial Institute
AEC	3-amino-9-ethylcarbazole
BCIP-NBT	5-bromo-4-chloro-3-indolylphosphate–nitroblue tetrazolium
ELISA	Enzyme-linked immunosorbent assay
ELISPOT	Enzyme Linked Immunospot
TIgA	Total immunoglobulin A
TIgG	Total immunoglobulin G
TcpA	Toxin-coregulated pilus
