# Supplementary material for: Evaluation of Cholera Antigen-Specific Gut-Homing β7-Positive Antibody-Secreting Cells in the Systemic Circulation of Oral Cholera Vaccinees Receiving Doses at Different Intervals
Source: Vaccines (Basel). 2025 Aug 28;13(9):919. doi: 10.3390/vaccines13090919 (PMC12474143; doi:10.3390/vaccines13090919)
Supplement: Supplementary file 1 [file vaccines-13-00919-s001.zip › vaccines-3690366-supplementary.pdf]

Supplementary file

Evaluation of cholera antigen-specific gut-homing β7-positive Antibody-Secreting Cells in the systemic circulation of Oral Cholera Vaccinees receiving doses at different intervals

Polash Chandra Karmakar [1], Rasheduzzaman Rashu [1], Mohammad Rubel Hoq [1], Umme Salma [1], Kamrul Islam [1], Nusrat Jahan [1], Naoshin Sharmin Nishat [1], Aklima Akter [1], Sultana Rownok Jahan [1], Pinki Dash [1], Amit Saha [1], Edward T. Ryan [2,3,4,‡], Firdausi Qadri [1,‡] and Taufiqur Rahman Bhuiyan [1*]

[1] icddr,b (International Centre for Diarrheal Disease Research, Bangladesh), Dhaka, Bangladesh
[2] Division of Infectious Diseases, Massachusetts General Hospital, Boston, Massachusetts, USA
[3] Department of Medicine, Harvard Medical School, Boston, Massachusetts, USA
[4] Department of Immunology and Infectious Diseases, Harvard School of Public Health, Boston, Massachusetts, USA
* Correspondence: taufiqur@icddrb.org

‡ Joint senior author on this work

Supplementary Figures

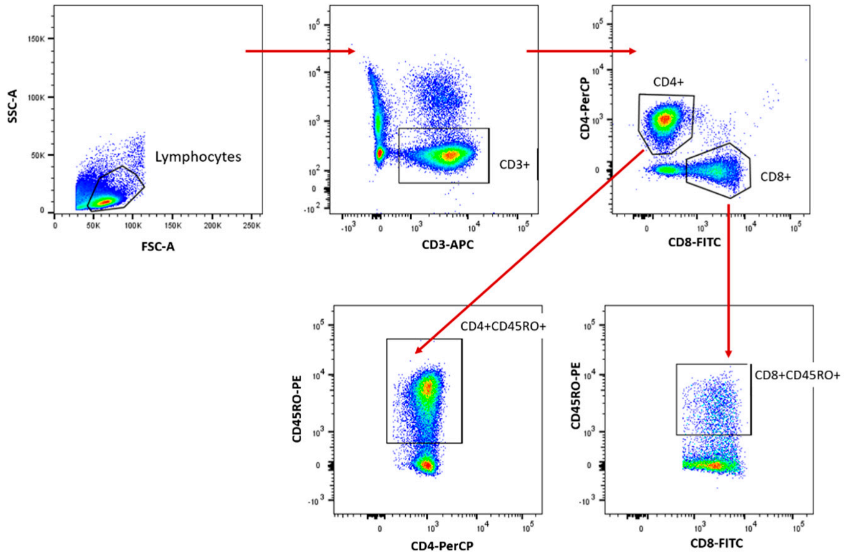

Supplementary Figure S1. Representative plot of fluorescence-activated cell sorting and gating strategy for memory CD4 and CD8 T lymphocytes.

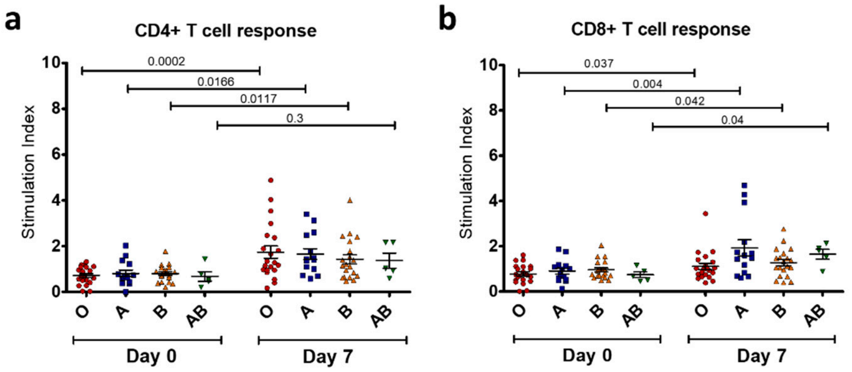

Supplementary Figure S2. Blood group-specific Helper (CD4+) and Cytotoxic (CD8+) T lymphocyte response in Shanchol vaccinees. Stimulation of T lymphocytes to *V. cholerae* membrane protein (AKI-MP) at D0 and D7 were evaluated separately according to the blood groups. Data from day 7 of all three vaccine groups shows the effects of the 1st dose of vaccine (considering single dose, two doses with a 14-day interval, and two doses with a 30-day interval). (a) CD4+ T cell response and (b) CD8+ T cell response. The letter “O”, “A”, “B” and “AB” in X axis indicate the ABO blood types. The responsive cell number after antigen stimulation, with its corresponding cell number to non-stimulation, indicates the stimulation index. Error bars show

standard errors of the mean (mean ± SEM). P ≤ 0.05 represents statistically significant differences from baseline levels (D0).

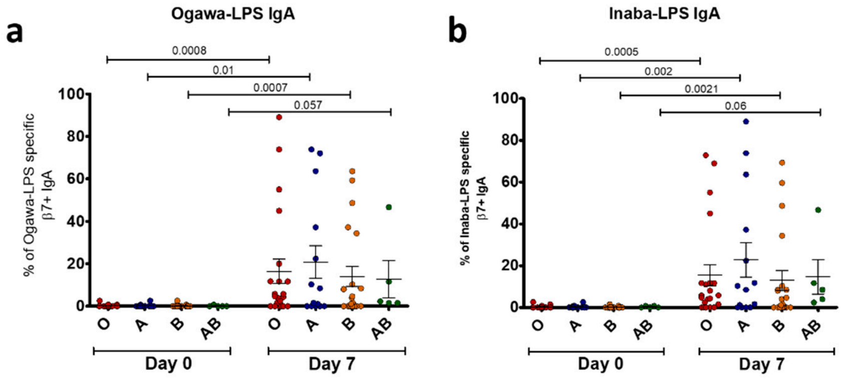

Supplementary Figure S3. LPS-specific gut-homing antibody-secreting cell (ASC) responses in Shanchol vaccinees among different blood groups. Stimulation of gut-homing (β7+) specific IgA immunoglobulin represents the effect separately according to the blood groups of vaccinees. Data from D7 of all three vaccine groups shows the effects of the 1st dose of vaccine (considering single dose, two doses with a 14-day interval, and two doses with a 30-day interval). (a) Ogawa-specific IgA (Ogawa-LPS IgA) and (b) Inaba-specific IgA (Inaba-LPS IgA). The letter "O", "A", "B" and "AB" in X axis indicate the ABO blood types. The responsive cell number after antigen stimulation, with its corresponding cell number to non-stimulation, indicates the stimulation index. Error bars show standard errors of the means (mean ± SEM). P ≤ 0.05 represents statistically significant differences from baseline levels (D0).

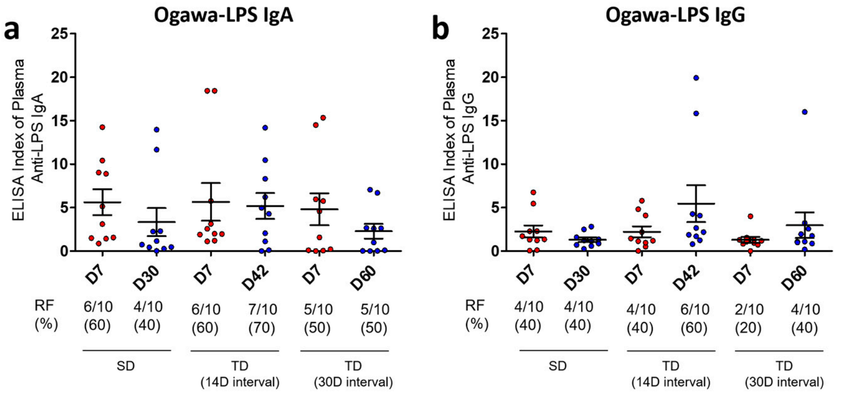

Supplementary Figure S4. LPS-specific Immunoglobulin A (IgA) and Immunoglobulin G (IgG) response to Ogawa in plasma. LPS-specific IgA (a) and IgG (b) responses among the three vaccine groups are plotted, where each single dot indicates the LPS antibody titer of an individual. The words SD and 2D mean single

dose and two doses, respectively. Two-tailed unpaired t-test was used for each of the vaccine groups at day 7 from 1st dose and one month after 2nd dose of vaccination. Horizontal bars represent geometric means (GM) and error bars indicate 95% confidence intervals. Responder frequencies (RF) and their percentages are mentioned.
